# Multiple Neuritis

**Published:** 1888-10

**Authors:** W. B. Hardman

**Affiliations:** Harmony Grove, Ga.


### MULTIPLE NEURITIS.

*Editors Atlanta Medical and Surgical Journal :*

I desire to report a case of multiple neuritis which I had the pleasure of seeing in company with my brother, Dr. L. G. Hardman, whose patient it was, and who asked me to report the case for him. This, I think, is the second case of the kind reported in the State, if this be a case, and I think there is but little grounds for saying that it is not, although it terminated fatally and we were not permitted to verify the diagnosis by an autopsy. The other case to which I refer is that of Dr. T. M. Holmes', of Rome, which my brother had the privilege of seeing at the late meeting of the Medical Association of Georgia.

Our patient was a woman, married, twenty-two years of age, of fair complexion, robust and healthy, though she had complained of headaches and slight dysmenorrhœa before her marriage. Some of her sisters also complain frequently of neuralgia and one has had dysmenorrhœa. With this exception her family history is good. She had had no other trouble up to the time of her final illness, save one attack of chronic catarrh of the cervix, which occurred soon after her marriage and lasted her about a year. But this was completely cured about six months before her neuritis came on.

The case did not come under our observation until it had progressed about eight weeks. But from the patient and her husband we gained the following history: Her hygienic surroundings were good, and there was no clue to any lead or arsenical poisoning. She had been perfectly well up to a certain Sunday night in February, when she rode a few miles to church in a buggy in company with her husband. The weather was damp and disagreeable, and when she returned she began to feel some lassitude and slight aching in her muscles, and bones also, seemingly. This continued to grow worse four or five days, until she became so weak that shs was compelled to take her bed. The pain in her muscles had grown more severe, and there was marked tenderness on touch, not increased by pressure.

She complained of tingling in her hands and feet, or the so-called "pins and needles." She had some headache. We were not able to learn the exact condition of her temperature and pulse at this time. But it was very high. The physician in charge gave her calomel, etc., for so-called "biliousness" and malarial fever, but she continued to grow worse; nausea and vomiting came on, and she retained but little food on her stomach. The pain and tenderness increased to such an extent that rheumatism was suspected, and she was given salicylate of sodium, not in large quantities, although there was no tenderness or swelling about the joints, but in the muscles between the joints, and other parts of the body. The physician in charge, nor the patient's friends, had noticed no swelling about the muscles or puffiness about the face up to this time. In this condition she came under my brother's care at the end of her eighth week of illness. When we saw her she was unable to sit up at all, or even hold her head up. She had been brought about fifty miles on the train, but this did not seem to worry her much. In fact, her husband seemed to think she was the better for it.

We had seen the patient several times before this illness, my brother having treated her for cervical catarrh, and the most striking symptoms on inspection were her marked, bleared eyes, the atrophy of the muscles on the back of her hands, and the swelling of her face and legs, things which the husband and attendants had not noticed before, perhaps from the fact that they were with her continually and the symptoms coming on gradually. She had been taking no arsenic. The muscular tenderness was so great at many points on her body that the slightest touch would make her cry out with pain; but there were some few spots of anæsthesia. Her temperature was not high, and never at any time during our treatment did it go above 101° F. Profuse sweating was noticed at times. Her pulse was weak, and beat about 120 to 130 to the minute from the time we saw her up to the time of her death, which occurred a little over three weeks after she came under our care. Her urine was examined, both chemically and microscopically, and there was no albumen or cast. Her bowels were costive and her physician had been giving her blue pills to move them. Her speech was slow and inarticulate, showing an involvement of the hypoglossal nerves. She had grown very childish and could not be reasoned with as formerly.

It had been my good fortune to hear the original Middleton Goldsmith Lecture on multiple neuritis, delivered by M. Allen Starr, under the direction of the New York Pathological Society, and to see a case or two demonstrated by him in his clinic, at the College of Physicians and Surgeons, New York, and some of his main points of differential diagnosis between this disease and polio myelitis and compression myelitis, we applied in this case with satisfactory results. Below I give a tabular statement of his most important points:

| POLIO MYELITIS. | MULTIPLE NEURITIS. |
|---|---|
| 1. Onset—acute or chronic. | 1. Sub-acute onset. |
| 2. In most cases one extremity is effected before the other and more local. | 2. In both extremities nearly at the same time. |
| 3. ncontinence of urine and fæces. | 3. No bladder or rectal disturbances. |
| 4 Girdle sensation common. | 4. Not present. |
| 5. Spasm of muscles seldom. | 5. Often found. |
| 6. Reaction, degeneration. | 6. The same. |
| 7. Paralysis and muscular atrophy. | 7. The same. |
| 8. Dropped foot seldom. | 8. Very common. |
| 9. Loss of tendon reflex. | 9. Slow and sluggish (?) and sometimes absent. |
| 10. No tenderness in the muscles. | 10. One of the most important signs. |
| 11. No tenderness along the nerve. | 11. Depends upon when you see the case. Often found. |
| 12. Few vaso-motor symptoms. | 12. More vaso-motor symptoms. |
| 13. Bed-sores common. | 13. No bed-sores. |

In this patient, as is observed from her history, the onset was *sub-acute*, and no particular limb was affected first, but the whole of her body at about the same time. But Prof. James Stewart, of Montreal, Canada, in Buck's Reference Handbook, speaks of the onset of multiple neuritis as being very acute as a rule, and "with the general symptoms of an acute infectious disease," yet he says it may be sub-acute.

Professor Francis T. Miles, of the University of Maryland, in

Pepper's System of Medicine, says: "Severe spontaneous, paroxysmal pains of a shooting, tearing character usher in the most of the cases." Our patient had no such pains in the beginning of her illness, but they became very severe later on. No bladder or rectal disturbances, and no girdle sensation. Marked clonic muscular spasms were noticed a few times after she came under our treatment, which extended over the whole of her body and continued for some little time. The electric currents showed the following characteristic signs: On the flexor surfaces of the forearm and arm there was slightly diminished Faradic contractility, and the galvanic current showed the C A C C only slightiy greater, and at some points only equal to the A N C C. The extensors showed little or no response to the Faradic current, and the A N C C markedly greater than the C A C C. She had no decided dropped foot or wrist, but there was more or less paresis of the whole body. The tendon reflex was absent, but the reflexes on the face and some other parts of the body were exaggerated. We could not test for ankle clonis on account of the great pain caused in the muscles of the leg. I have spoken of the marked muscular tenderness that this case presented, and this Starr considers one of the most important signs of diagnosis. He says he has never seen it absent. There was decided tenderness along some of the nerves, but not along all of them. There was tenderness along the intercostal nerves, but this ceased just before reaching the spine. No bedsores appeared.

*The causes* of this disease, as given by various authors, are many. It is spoken of by some as being due to some morbid constitutional affection, and they support this by reference to the Japanese kakke or Indian beri-beri, which has the same characteristic pathological changes and symptoms in common with this disease. Tuberculosis, syphilis, diphtheria, typhoid fever, measles, small-pox, etc., are spoken of as exciting causes, but all of these can be excluded in this case. It has also been known to occur at times with polio arthritis. Of poisons, lead, arsenic, alcohol, ergot, copper, etc., will produce it, but they cannot be connected with this case. Riding in a jolting wagon, it is said, has caused it.

When no other cause can be found, most authors attribute it to cold and exposure, and in this case there seems to be no other way of explaining it.

*The pathology* of multiple neuritis has been fully discussed by M. Allen Starr in his Middleton Goldsmith Lectures, and he holds it to be a nerve degeneration, "corresponding quite exactly" to that artificially produced by experiments on animals.

*Wallerian Degeneration.*—The danger of death from this trouble lies at the onset from increased heart action, due to the degeneration of the pneumogastric nerve. This our patient escaped in the beginning, but succumbed finally, perhaps, to the same cause. After this danger has been avoided for the first four weeks of the disease, most authors speak of recovery as almost certain. Starr relates a case in which not a muscle in the body could be moved, except the diaphragm, and the patient recovered. *The treatment* prescribed by different writers is to remove the cause if possible, some recommend salicylic acid or salyclate of soda in large doses, iodide of potash, etc., while others use tonics, strychnine, arsenic, etc, and later electricity in two ways, viz., the galvanic current in the muscles and also through the nerves. The treatment used in this case was as follows: The salicylate of soda and the other medicines she had been taking were discontinued, as these were supposed to be the cause of her nausea and vomiting and not the disease itself. She was then put on four drop-doses of Fowler's solution of arsenic, given in soda mint, and hypodermic injections of morphine were directed to be used when needed for her pain and restlessness, but she did not have to use it but three or four times during the three weeks she was under our care. Before, she had been using blue pills to keep her bowels moved, and her physician had been giving her large doses of chlorodyne and chloral to make her rest. As soon as we began the arsenic and stopped the other drugs, her nausea and vomiting stopped at once, her bowels moved regularly and she began to improve. She soon got so she could eat with a relish, a thing she had not done in quite a while before. But after a week or more she came to a standstill, yet the last time we saw her she seemed to be doing very well indeed. I do not know the exact manner in which she died, as no physician was present, death coming on somewhat suddenly, which would suggest the heart or respiratory apparatus as the cause. Electricity was not used for therapeutic purposes, though we intended to begin it the week she died.

It may be well enough to remark that she was not pregnant.

*Harmony Grove, Ga., Aug. 2, 1888.* W. B. HARDMAN.

